# Mycobacteriophages in diagnosis and alternative treatment of mycobacterial infections

**DOI:** 10.3389/fmicb.2023.1277178

**Published:** 2023-09-28

**Authors:** Xudong Ouyang, Xiaotian Li, Jinmiao Song, Hui Wang, Shuxian Wang, Ren Fang, Zhaoli Li, Ningning Song

**Affiliations:** ^1^School of Life Science and Technology, Weifang Medical University, Weifang, China; ^2^Weifang Key Laboratory of Respiratory Tract Pathogens and Drug Therapy, Weifang, China; ^3^SAFE Pharmaceutical Technology Co. Ltd., Beijing, China

**Keywords:** mycobacteriophage, mycobacterial infection, non-tuberculous mycobacteria, phage therapy, *Mycobacterium tuberculosis*

## Abstract

Antimicrobial resistance is an increasing threat to human populations. The emergence of multidrug-resistant “superbugs” in mycobacterial infections has further complicated the processes of curing patients, thereby resulting in high morbidity and mortality. Early diagnosis and alternative treatment are important for improving the success and cure rates associated with mycobacterial infections and the use of mycobacteriophages is a potentially good option. Since each bacteriophage has its own host range, mycobacteriophages have the capacity to detect specific mycobacterial isolates. The bacteriolysis properties of mycobacteriophages make them more attractive when it comes to treating infectious diseases. In fact, they have been clinically applied in Eastern Europe for several decades. Therefore, mycobacteriophages can also treat mycobacteria infections. This review explores the potential clinical applications of mycobacteriophages, including phage-based diagnosis and phage therapy in mycobacterial infections. Furthermore, this review summarizes the current difficulties in phage therapy, providing insights into new treatment strategies against drug-resistant mycobacteria.

## 1. Introduction

Antimicrobial resistance has been one of the major threats to human health (Hancock, 2005; Dcosta et al., 2011; Ling et al., 2015; Adedeji, 2016; Holmes et al., 2016; Hoeksma et al., 2019; Ouyang et al., 2022). The resistant bacteria, especially multidrug-resistant “superbugs,” have led to higher patient mortality and treatment costs (Butler and Buss, 2006; Gelband et al., 2015; Ouyang et al., 2021). The same applies to mycobacterial infections. *Mycobacterium tuberculosis* (MTB) and other non-tuberculous mycobacteria (NTM) are responsible for some of the most difficult-to-treat infections that are a threat to millions of humans globally (Shield et al., 2021). Tuberculosis (TB), which is caused by the MTB complex, is one of the oldest communicable diseases that continues to be a major cause for concern. In fact, TB was the leading global cause of death until the coronavirus pandemic, ranking even above HIV/AIDS (World Health Organization, 2022b). In 2021, there were approximately ten million people who developed TB worldwide, resulting in 1.4 million deaths among HIV-negative people and an additional 187,000 among HIV-positive people (World Health Organization, 2022b). Although TB is treatable, the emergence and spread of drug-resistance to first-line TB drugs like isoniazid and rifampicin is gradually making the disease relatively incurable (Seung et al., 2015). The MTB isolates that are resistant to these two drugs are called multidrug-resistant (MDR) MTB. The existence of these resistant strains can result in treatment failure when standard first-line treatments are used. Although there are second-line medicines available on the market, these drugs are usually toxic and have adverse side effects (World Health Organization, 2022a).

Opportunistic mycobacterial infections are caused by NTM, which include other mycobacterial species, other than *Mycobacterium leprae* and MTB (Kumar and Kon, 2021). The most common human NTM pathogens are the slow growing *Mycobacterium avium* complex (MAC), *Mycobacterium xenopi*, *Mycobacterium fortuitum* complex, *Mycobacterium kansasii*, and the rapidly growing *Mycobacterium abscessus* group (MABS) (Ratnatunga et al., 2020). Recently, NTM diseases gained increasing attention owing to their chronic debilitating properties, along with substantial morbidity and mortality (Donohue and Wymer, 2016; Thomson et al., 2020; Winthrop et al., 2020). Unlike *M. tuberculosis*, NTM are ubiquitous organisms with generally intrinsic antibiotic resistance that can withstand a variety of environmental conditions, which partly explains why NTM diseases are difficult to diagnose or treat (Ratnatunga et al., 2020). Because of the high intrinsic and/or acquired antimicrobial resistance levels associated with NTM and TB diseases, the need for new strategies to treat mycobacterial infections cannot be overstated. Therefore, many alternative approaches have been developed and introduced to combat antimicrobial resistance, and these include bacteriophage therapy, which is quite promising.

Bacteriophages, or phages, are viruses that selectively infect bacteria and have either narrow or broad host ranges. They are generally classified into two forms, which are lytic or lysogenic, mainly based on how they infect host cells. Lytic phages infect bacterial cells, using their machinery to replicate themselves, before destroying the host cells. On the other hand, temperate phages can choose from a lysogenic or lytic life cycle. For a lysogenic mode, phages integrate their genetic material into the host chromosome, and repress lytic gene expression prior to switching to a lytic life mode (Puiu and Julius, 2019). Using phages to lyse bacteria that cause infectious diseases is generally not a new anti-infection treatment approach though there has been a renewed focus on application in antimicrobial resistance (Abedon et al., 2011). Recently, phage therapy was reported to successfully cure a 15-year-old girl who was suffering from a disseminated *M. abscessus* infection post-lung transplantation (Dedrick et al., 2019). In another case, a 26-year-old man who suffered from severe cystic fibrosis with chronic *M. abscessus* pulmonary infection was also reportedly cured through phage therapy (Nick et al., 2022). These and other reports shed light on a new possible way of treating mycobacterial infections. The purpose of this review is to provide insights into potential phage applications against mycobacterial infections in modern healthcare. In addition to a brief introduction to the phages that specifically target mycobacteria, which are mycobacteriophages, we will mainly focus on their use as diagnostic tools and as an alternative treatment approach.

## 2. Mycobacteriophages

Mycobacteriophages are a diverse group of bacteriophages that specifically infect mycobacteria as their hosts. They are among the most abundant organisms on earth and can be isolated from soil, water, sewerage and any other environment that has been colonized by mycobacteria (McNerney and Traore, 2005). Mycobacteriophages were first found from soil and leaf mould in 1946 (Gardner and Weiser, 1947), and more than 12,000 individual mycobacteriophages have been identified and classified to date.^1^ The first mycobacteriophage genome was sequenced in the 1990s (Hatfult and Sarkis, 1993) and over 2,000 strains have been sequenced by the year 2023. As the number of successfully sequenced genomes increases, it is clear that some phages are closely related, based on the similarities in their overall nucleotide sequences, so they are grouped into “clusters” (Puiu and Julius, 2019). Phages from the same cluster are expected to share significant similarities in their nucleotide sequences. Little to no similarity should be observed in phages that belong to different clusters. Phages that have no similarity to any other strains are regarded as singletons, and seven have, so far, been excluded from a total of 31 clusters (Table 1). Mycobacteriophage clusters are designated Clusters A–Z and Clusters AA–AE, with Cluster A containing the largest number of strains (703 members) while Cluster AE, X, and Z each consist of only two strains. For phages that are recognizably distinguished within a cluster, boundaries were defined to separate them into subclusters, mainly based on their average nucleotide identity (ANI) values (Hatfull, 2012). As shown in Table 1, 12 out of 31 clusters are divided into subclusters. Cluster A is the most diverse cluster with 20 subclusters, 19 of which infect the host of *Mycobacterium*, while one subcluster (A15) infect *Gordonia* (data from A15 is excluded in Table 1). Interestingly, though Cluster E is the 6th largest cluster with 114 individual members, it is not divided into any subcluster. This is because there are only some small changes in a single gene or a small group of genes, which are far from identical (Hatfull, 2018). Phages from the same cluster generally have shared features, such as life cycle, repeated sequences, and regulatory systems, but the relationship between different phage isolates is complex and difficult to illustrate. This is due to the architectural mosaicism of phage genomes, most of which are just an assembly of single gene modules (Pope et al., 2015). In many cases, the adjacent genes of a shared gene in different genomes can be distinct, but the clear mechanisms that bring the genome mosaicism are yet to be determined (Hatfull, 2022). Morphologically, mycobacteriophages are tailed dsDNA viruses with myoviral or siphoviral morphotypes. The myoviral mycobacteriophages have contractile tails, and they all belong to a single genomic cluster, which is, Cluster C. Although the other mycobacteriophages have notable genomic diversity, they all have siphoviral morphologies, meaning that they have long flexible non-contractile tails. Among the identified mycobacteriophages, there are no podoviral morphotypes, presumably due to the physical barrier presented by the thick mycobacterial cell wall that contains glycolipids, mycolic acids, and glycopeptidolipids (Hatfull, 2018).

**Table 1:** Clusters and subclusters of mycobacteriophages^*^.

Cluster	Number of subclusters/Name of the singleton strain	Number of members	Average length (bp)	Average GC%
A^**^	19	704	51,607	63.4
AA	--	2	140,785	67.4
AB	--	5	49,672	58.6
AC	--	4	70,029	49.8
AD	--	3	64,480	66
AE	--	2	71,497	58.8
B	13	362	68,791	67.1
C	2	163	155,555	64.7
D	2	21	64,805	59.6
E	--	114	75,542	63
F	5	203	57,326	61.5
G	5	65	42,304	66.9
H	2	10	69,108	57.4
I	2	7	50,320	66.4
J	--	38	111,065	60.9
K	7	165	59,929	66.8
L	5	66	74,479	58.8
M	3	15	81,869	61.2
N	--	38	42,905	66.2
O	--	21	71,287	65.4
P	6	43	47,917	67
Q	--	20	53,844	67.4
R	--	8	71,339	56
S	--	17	64,993	63.4
T	--	7	42,746	66.2
U	--	3	66,864	50.4
V	--	4	77,869	57
W	--	6	61,013	67.5
X	--	2	88,037	56.7
Y	--	4	76,836	66.7
Z	--	2	50,807	66
Singletons	DS6A	1	60,588	68.4
	Identity Crisis	1	38,341	65
	Kumao	1	70,373	62.1
	Lil Spotty	1	49,798	64.7
	Malagasy Rose	1	54,301	65.3
	MooMoo	1	55,178	62
	Sparky	1	63,334	65.2

^*^Data were obtained from Actinobacteriophage database (phagesdb.org).

^**^Subcluster A15 is excluded from the calculation of Cluster A, because its host is Gordonia.

Due to the increasing importance of treating mycobacterial diseases, mycobacteriophages have since been isolated and studied from the late 1940s (Hatfull, 2010). The main foci of the early work were on the characterization of new phages and on their application as typing tools in identifying clinical mycobacterial isolates. This remained so until the 1970s. However, in the following two decades, the massive success of antibiotics slowed down the research on mycobacteriophages (Hatfull, 2012). This situation changed in response to the development of molecular genetic biology and the spread of antimicrobial resistance. The resurgence of mycobacteriophage studies started in the late 1980s (Jacobs et al., 1989), as interest in their molecular biology arose, along with their exploitation as gene manipulation tools for mycobacteria arose (Jacobs et al., 1987; Lazraq et al., 1991; McNerney, 1999; Bardarov et al., 2002; Marinelli et al., 2008; Tufariello et al., 2014; Hatfull, 2018; Murphy et al., 2018; Wetzel et al., 2021).

## 3. Diagnosis

Mycobacterial infections, including multidrug-resistant (MDR) or extensively drug-resistant (XDR) strain infections, are treatable diseases. However, the patients should be properly treated from the early stages of infection for interventions to be successful. This means that the diagnosis should be quick and effective, to reduce morbidity and mortality (Dominguez et al., 2015). Currently, several diagnostic approaches for mycobacteria are available worldwide. The gold standard is the traditional mycobacterial culture method, which has been used for decades, and is still relevant today due to its low cost and high sensitivity. However, since many mycobacterial species are slow growing, detection may require several weeks. For example, the TB pathogen MTB can take up to 12 weeks to grow before its recognition on plates (Shield et al., 2021). A faster approach would be sputum smear microscopy, which is another widely used method with lower costs. However, the sensitivity of this approach is relatively low. Moreover, it has a weak ability to distinguish dead cells from viable ones. Some improvements have been put in place to overcome these limitations while keeping this method still affordable in middle- and low-income countries. These include optimizing the sample preparation and updating the microscope system (Chandra et al., 2016; Shah et al., 2016; Ojha et al., 2020), though even with such improvements, the culturing method remains more sensitive. Molecular platforms have provided some advanced approaches, such as real-time PCR (RT-PCR), enzyme linked immunosorbent assay (ELISA), Clustered Regularly Interspaced Short Palindromic Repeats (CRISPR)-based methods, and biosensor assays (Helb et al., 2010; Wadhwa et al., 2012; Dominguez et al., 2015; Ai et al., 2019; Osei Sekyere et al., 2019; Lyu et al., 2020). For example, the popular RT-PCR platform GeneXpert has comparable sensitivity to the culturing method, but only takes a few hours to deliver results (Helb et al., 2010; Osei Sekyere et al., 2019). Nevertheless, the complicated equipment that is required to perform these molecular assays, in addition to the need for trained personnel and the high costs cannot be ignored. Even though these methods are rapid and sensitive, they are unaffordable in the high TB-burden countries, most of which are characterized by low- and middle-income.

Phage-based strategies are a relatively new addition to the mycobacteria diagnostic toolbox. Due to the specific binding of phages to host bacteria, mycobacteriophages have the potential to detect mycobacterial strains in a manner that is more sensitive and specific. In principle, culturing mycobacteriophages does not require sophisticated laboratory equipment, thus the cost of phage-based strategies is affordable for the high TB-burden countries. The phage-based diagnostic strategies were generally developed in two directions, either relying on the phage amplification properties or using genetically modified reporter phages (Fu et al., 2015; Shield et al., 2021). We will discuss both strategies in this section.

### 3.1. Phage amplified biologically (PhaB) assay

**Figure 1:**
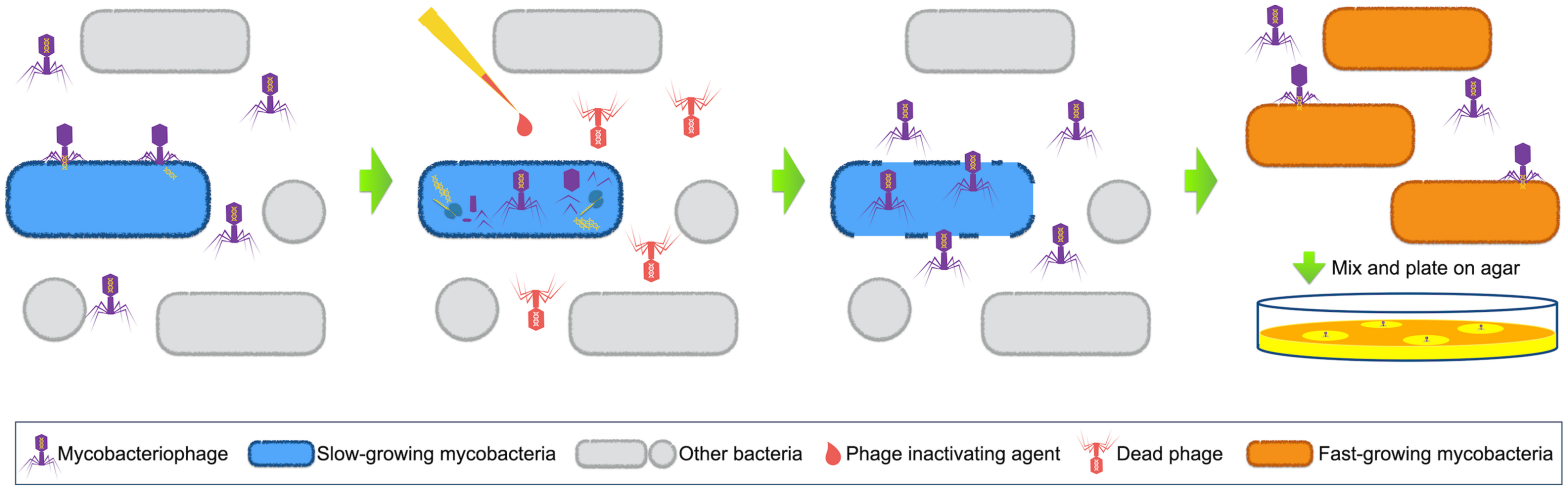
Schematic representation of phage amplified biologically assay. Mycobacteriophages are used to infect patient samples. When mycobacterial pathogens (normally slow-growing mycobacteria) exist, phages will insert their DNA into these host cells and start to replicate. Phage inactivating agents are afterwards added to destroy all the non-infected exogenous phages. The phage inactivating agents are then removed, and the newly released phages are collected to mix with sensor cells (fast-growing mycobacterial cells). Next, the mixture is plated on agar and plaques are checked after incubation.

Wilson et al. introduced the concept of the PhaB assay in 1997 by establishing a rapid method for testing the drug susceptibility of MTB using the D29 phage (Wilson et al., 1997). D29 is a lytic, double-stranded DNA phage from Subcluster A2 in Cluster A. It has a broad host range of *Mycobacterium* spp., which include *M. tuberculosis*, *M. bovis*, *M. avium*, and *M. smegmatis*. The principle behind this method is based on the fact that live bacterial hosts can protect infected phages from inactivation by virucides, and the procedure is depicted in Figure 1 (Wilson et al., 1997; McNerney et al., 2004; Símboli et al., 2005). Basically, D29 phages are used to infect patient samples that might contain the slow-growing MTB cells. The infected phages are protected by bacteria and will replicate within the host cells, while the non-infected exogenous phages are inactivated by an added phage inactivating agent. The phage inactivating agent is then removed, before the replicated phages break the host cells and are released. Next, the released phages are mixed with a fast-growing mycobacterium, which should also be a host for the D29 phage. The plates are incubated for a certain period of time, after which plaques are checked.

Based on the PhaB assay, a commercial phage-based kit, FASTPlaqueTB™, was developed by Biotech Labs Ltd. (Ipswich, United Kingdom) (Beinhauerova and Slana, 2021). The sensitivity and specificity of FASTPlaqueTB™ are 87.5 and 96.9%, respectively, similar to the PCR detection method (Albay et al., 2003). Subsequently, more variants of this kit were designed to determine drug resistance in human samples, such as FASTPlaqueTB-RIF™, FASTPlaqueTB-MDR™ and FASTPlaqueTB-Response™ (Albert et al., 2001; Beinhauerova and Slana, 2021). The principle of these resistance-detection kits was similar to the original FASTPlaqueTB™ kit, except that bacteria were inactivated, and host protection was eliminated by adding antibiotic agents, before the initial phage infection step. Therefore, if the host mycobacteria are not drug-resistant, no active phages would be detected in the end. The timing of addition of antibiotics is critical to an accurate diagnosis. Since antibiotics with different mechanisms of action (MOA) need different time to inhibit the growth of bacterial cells, it is recommended to set different time depending on the particular antibiotic. The in-house variants of these kits are commonly used as a suitable alternative to molecular methods in low- and middle- income countries, due to their high sensitivity and low cost. In addition, although these kits were originally developed for the diagnosis of human TB, they were also successfully customized to detect other mycobacteria (Altic et al., 2007; Foddai et al., 2009).

However, since the D29 phage has a broad host range, it is unable to completely confirm the specific species of mycobacteria. To address this limitation, the PCR procedure could be subsequently carried out after the PhaB assay. Stanley et al. achieved this by adding a series of steps after PhaB, including plaque harvesting, DNA extraction, and plaque PCR, and termed this method the Phage-PCR assay (Stanley et al., 2007). The Phage-PCR assay was later optimized and marketed as the Actiphage® (PBD Biotech Limited, Thurston, Suffolk, United Kingdom) (Swift et al., 2020). This updated approach does not require the detection of plaques by lawns of fast-growing mycobacteria. Instead, the mycobacterial DNA that was released from the liquid that was lysed by bacteriophages is filtered and purified, followed by direct detection using PCR. This significantly reduced the time taken to deliver results (generally within 6–8 h) (Swift et al., 2020). In addition, this approach has enhanced sensitivity, considering that there are fewer chances for sample loss.

Other modifications of the PhaB assay include adding a peptide-mediated magnetic separation (PMS) step to selectively separate the target bacterial cells from other cells (Foddai et al., 2010). Also, the D29-specific ELISA assay could be used after phage amplification, instead of the plaque assay (Stewart et al., 2013). The PMS can strongly enhance the analytical specificity and sensitivity of the subsequent test, because it reduces the background noise, and concentrates the target cells to a smaller volume as well (Foddai et al., 2010, 2011). With PMS, Foddai et al. achieved 85–100% capture of their target bacteria and less than 1% capture of other *Mycobacterium* spp., a scenario that increased the sensitivity of the subsequent phage amplification assay to 0.3 PFU/mL (Foddai et al., 2010). Later on, Stewart et al. coupled the PMS-phage assay with a novel phage detection method using a polyclonal antibody produced against the D29 phage (Stewart et al., 2013). Although no further research on the PMS-phage-ELISA assay was reported, this assay is a promising detection method for viable mycobacteria. Despite the fact that these two modified PhaB assays were originally developed for detecting *Mycobacterium avium* subsp. *paratuberculosis* in milk and feces from cattle, they are also potentially novel approaches for the diagnosis of NTM infectious diseases. Compared to the original PhaB assay, the modified assays may be associated with higher costs. However, the development of these approaches may eventually lead to point-of-care diagnostics that are based on phage, possibly benefiting the whole world, including the high-income countries.

### 3.2. Phage reporter assays

**Figure 2:**
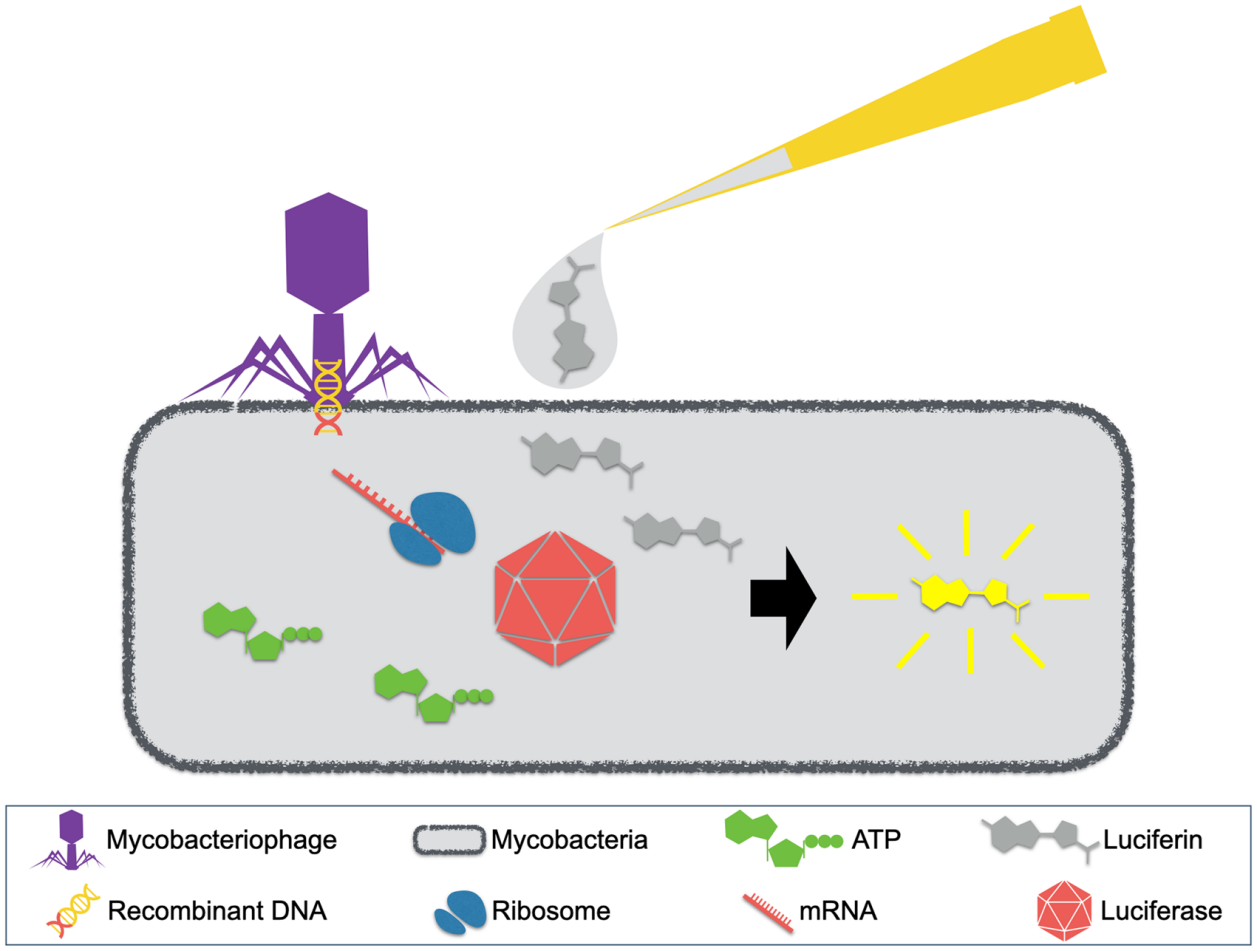
Luciferase reporter phage (LRP) assay. LRPs, mycobacteriophages with recombinant DNA containing the firefly luciferase gene, are used to infect patient samples. When mycobacterial pathogens exist, luciferse will be expressed. With the presence of ATP and exogenously added luciferin, luminescence will be generated.

The phage reporter assays were originally designed with luciferase reporter phages (LRPs). These LRPs are recombinant mycobacteriophages that carry the firefly luciferase gene and they can be applied in the detection of mycobacterial strains. The concept of LRP assays is depicted in Figure 2 (Jacobs et al., 1993; Kalantri et al., 2005; Fu et al., 2015). Generally, LRPs insert the recombinant DNA fragment containing the firefly luciferase gene into their host cells, which then express the luciferase. In the presence of adenosine triphosphate (ATP) and exogenously added luciferin, luminescence will be generated and detected. Notably, luminescence requires available ATP that is provided by viable host cells, which could be inhibited by antimycobacterial agents. Therefore, drugs that inhibit host metabolism will inhibit the synthesis of ATP and the expression of luciferase, resulting in the absence of detectable light (Jacobs et al., 1993).

The first-generation LRP phAE40 was derived from phage TM4 in 1993 and is only capable of detecting at least 10^4^ cells/mL (Jacobs et al., 1993). Although it has the advantage of being less time-consuming than culturing, LRP phAE40 only provides proof of concept due to its limited sensitivity and will, therefore, require further development/optimization (Jain et al., 2011). Subsequently, LRPs were generated from other mycobacteriophage species, in a bid to overcome this shortfall. A temperate phage L5 derived LRP shows high sensitivity with *M. smegmatis*, which could be used as a rapid tool for screening antimycobacterial drugs. Unfortunately, this LRP is unable to target MTB (Sarkis et al., 1995). Another LRP that was derived from lytic phage D29 exhibited higher sensitivity compared to the first-generation phAE40, in addition to a broad host range that includes MTB (Pearson et al., 1996). However, several improvements are still required before the clinical application of this LRP (Dusthackeer et al., 2008). It is also important to note that LRPs generally require large numbers of cells to generate detectable luminescence, making it difficult to visualize single infected cells. Therefore, similar to the original PhaB assay, LRP assays are unable to determine the presence of multiple mycobacterial strains within one sample.

Another group of phage reporters is fluoromycobacteriophages, initially developed by Piuri et al. in 2009 (Piuri et al., 2009). Instead of the luciferase gene, *gfp* or *ZsYellow* genes were delivered by phages into host cells. This new feature affords fluoromycobacteriophages several advantages over LRPs. For example, the simplicity and sensitivity could improve, considering that fluoromycobacteriophages could be detected by either fluorescent microscopy or flow cytometry. Also, fewer than 100 cells/mL can be detected, and drug resistant cells can be distinguished within mixed cultures. Moreover, the emission of fluorescence does not require any exogenous substrates, not to mention that samples can be fixed by paraformaldehyde, which increases biosafety and facilitates sample transportation (Piuri et al., 2009). A second-generation fluoromycobacteriophage was recently developed using a modified *mCherry* gene with codon usage optimized for mycobacteria, and higher sensitivity and shorter detection time were achieved (Urdániz et al., 2016). Although fluorescence microscopy or flow cytometry involves expensive equipment, the use of light-emitting diode (LED) fluorescence has partially solved this problem, making it well-suited for low- and middle-income countries (Piuri and Hatfull, 2019).

## 4. Treatment

Due to the increasing cases of MDR-MTB and MDR-NTM infections, treatment is becoming more complex and difficult. New treatment regimens are urgently needed to improve the efficiency and safety, reduce therapy duration, minimize permanent lung damage, and prevent further resistance development (Zumla and Abubakar, 2017). Phage therapy has gained increased attention, owing to its high specificity and relatively low toxicity. Phage therapy involves lysing the causative pathogen of a relevant infection by direct administration of live virulent phages to a patient. When approaching the surface of a host cell, the lytic phage recognizes the specific surface receptor using its receptor binding proteins (RBPs) which are at the tip of the phage tail (Kortright et al., 2019; Stone et al., 2019). When specific binding occurs, the nucleic acid of the phage is subsequently injected into the host cell and the phage replication process begins. Afterward, the newly assembled phage particles will lyse the host cell, release themselves into the surrounding environment, and continue to infect other bacterial cells. To achieve bacterial lysis, phage-encoded endolysin, which is a peptidoglycan degrading enzyme, is synthesized at the latest stage of phage gene expression. Through the small membrane pores that are generated by the protein holin, endolysins can reach their substrates and degrade the cell wall from the inside (Stone et al., 2019).

### 4.1. Therapeutic trials of phage therapy

The first phage therapy was performed in 1919 by d’Hérelle, who treated chickens that were infected by *Salmonella gallinarum* (Kortright et al., 2019). Later on, he attempted to use phages to treat human infectious diseases, and was successful in preventing an outbreak of cholera by introducing phages into drinking wells (D’Herelle, 1929). Other scientists were encouraged by these outcomes, and therefore, tried phage therapy for addressing many infectious diseases, though they failed to achieve consistent success. Afterward, the interests in phage therapy were replaced by attention toward antibiotics, leading to a pause of phage therapy in western medicine. Since the beginning of the 21st century, the world has rediscovered the therapeutic potential of phage therapy, mainly because of the emerging crisis of antimicrobial resistance. The safety and efficacy of phage therapy via different delivery routes (oral administration, topical administration, intravenous administration, intraoperative administration, and intrarectal administration) is largely determined by clinical trials (Jikia et al., 2005; Leszczyński et al., 2006; Letkiewicz et al., 2009; Ujmajuridze et al., 2018; Nir-Paz et al., 2019; Luong et al., 2020; Rubalskii et al., 2020).

Bruttin and Brüssow tested the safety of the orally administrated *Escherichia coli* phage T4 in 15 healthy adults (Bruttin and Brüssow, 2005). The results showed that the phage T4 neither induces T4-specific immune response, nor causes treatment-related adverse events, suggesting that phage therapy is safe for treating diarrheal diseases. In another clinical trial for phage therapy, oral treatment with phages resulted in the elimination of an methicillin-resistant *Staphylococcus aureus* (MRSA) strain that was colonized in the gastrointestinal and urinary tracts, demonstrating the success of orally administrated phage therapy (Leszczyński et al., 2006).

The topical route of administration is mostly applied in skin and ear infections. Wright et al. assessed the safety and the efficacy of a six-phage cocktail against antimicrobial resistant *Pseudomonas aeruginosa* colonized in 24 patients with chronic *otitis* (Wright et al., 2009). After a 42-day-course of phage therapy, no phage-related adverse events were observed, and the counts of *P. aeruginosa* were notably lower in the treated group than the control. In another case report, two patients with severe local radiation injuries were subsequently infected by a multidrug resistant *S. aureus*, which could not be eliminated during a 1 month hospitalization using various medications, including topical ointments and antibiotics (Jikia et al., 2005). Afterwards, PhagoBioDerm, which is a phage-antibiotic fused drug, was given to both patients and this was followed by rapid termination of purulent drainage.

The intravenous route for delivering phages is quite popular. In 2017, phage therapy was used in a two-year-old patient with multidrug-resistant *P. aeruginosa* infection in the blood, because the patient is allergic to multiple antibiotics (Duplessis et al., 2018). A cocktail containing two phages was intravenously administrated to the patient, which sterilized the patient’s blood after 4 weeks of continuous bacteremia. In a case in 2019, a patient with bacterial osteomyelitis associated with the multi-drug resistant *Klebsiella pneumoniae* and extensively drug resistant *Acinetobacter baumannii* was treated using a combination of phages and antibiotics (Nir-Paz et al., 2019). As a result, eradication of the infection and tissue healing were rapidly observed, and amputating the patient’s one leg was therefore unnecessary. Notably, a phage-resistant *A. baumannii* mutant developed during the treatment. The researchers quickly isolated a new phage for phage therapy to combat the new strain, indicating the flexibility of phage treatments (Nir-Paz et al., 2019). In other case reports, intravenous administration of phages was also used to treat lung infections, joint infections, skin infections, etc. (Khawaldeh et al., 2011; LaVergne et al., 2018; Aslam et al., 2019a,b; Doub et al., 2020). Elimination of causative pathogens was mostly observed and no severe phage related adverse events were ever determined.

In addition to these phage administration strategies discussed above, phages can be administrated intraoperatively as well. The inhalation and intrarectal routes are also popular against lung and urethra infections. Similarly, these routes are determined as safe and efficient administration strategies, showing high potential value in clinical application (Letkiewicz et al., 2009; Chan et al., 2018; Ujmajuridze et al., 2018; Rubalskii et al., 2020).

### 4.2. Phage therapy in mycobacterial infection

Limited case reports about phage therapy against mycobacterial infection are currently available (Table 2) (Allué-Guardia et al., 2021; Hatfull, 2023). However, the idea of using mycobacteriophages to treat patients has long been discovered. Back to the early 1980s, experiments had already been designed to assess the therapeutic effects of mycobacteriophages in guinea pigs with MTB infection (Sula et al., 1981). In the past few years, the development of novel biotechniques and experimental designing standard has seen phage therapy being improved to enhance its therapeutic efficacy and reduce the chance of phage resistance occurrence (Lenneman et al., 2021). Eventually, in 2019, the first case report on using phage therapy against human mycobacterial infection was published (Dedrick et al., 2019). The patient was a 15-year-old individual with cystic fibrosis and comorbidities, also suffering from chronic *P. aeruginosa* and *Mycobacterium abscessus* infections. The patient had been on anti-NTM therapy for eight years. The patient then underwent an uncomplicated bilateral lung transplant, followed by administration of immunosuppressive drugs and multiple antibiotics. However, antibiotic treatment had to be ceased due to some severe side effects, but bacterial infections recrudesced. Antibiotic therapy was resumed and continued with a palliative care plan, but more skin nodules appeared, and some areas of the surgical wound broke down, suggesting that the infection was not under control. Given this condition, phage therapy was applied under a compassionate use with a cocktail of three phages, including a natural phage Muddy and two genetically modified phages, BPs*33*ΔHTH-HRM10 and ZoeJΔ*45*. The phage cocktail was intravenously administrated every 12 h for at least 32 weeks, and this led to improvements in surgical wound, skin lesions, lung function, and liver function. Phage therapy was well tolerated during the whole treatment, with no reports of significant adverse reactions. Although weak antibody responses to phage proteins were detected, phage neutralization was, fortunately, not shown (Dedrick et al., 2019).

**Table 2:** Examples of phage therapy in mycobacterial infections.

Patients	Underlying conditions	Organisms	Phages used	Administration	Outcomes	References
A 15-year-old individual	Cystic fibrosis, bilateral lung transplant	*M. abscessus*	Muddy, BPs33ΔHTH-HRM10, ZoeJΔ45	Intravenous, topical	Reduced infection, improved organ functions	Dedrick et al. (2019)
20 patients with drug-resistant mycobacterial disease	Cystic fibrosis, scleroderma, arthritis, mendelian susceptibility to mycobacterial disease	*M. abscessus*, *M. chelonae*, *M. avium*, BCG	BPs∆33HTH_HRM10, Muddy, ZoeJ∆45, Itos, BPs∆33HTH_HRM^GD03^, D29_HRM^GD40^, Fionnbharth∆43∆45, Fred313cpm∆33	Intravenous, topical, chest wash, aerosol, bronchoscopy	11 patients had favorable or partial responses, 5 had inconclusive responses, and 4 had no evident clinical improvement	Dedrick et al. (2022) and Hatfull (2023)
An 81-year-old individual	Bronchiectasis	*M. abscessus*	Muddy, BPs33ΔHTH-HRM10, ZoeJΔ45	Intravenous	Reduced infection within the first month, but eventually failed due to neutralizing antibody response	Dedrick et al. (2021)
A 26-year-old individual	Cystic fibrosis, bronchiectasis	*M. abscessus*	BPsΔ33HTH_HRM10, D29_HRM^GD40^	Intravenous	Reduced infection	Nick et al. (2022)

This new-found success in phage therapy encouraged scientists in this field, and many related studies have been recently undertaken. Guerrero-Bustamante et al. screened phages against clinically isolated MTB strains, and subsequently modified these phages using phage genetic tools (Guerrero-Bustamante et al., 2021). As a result, these researchers assembled a five-phage cocktail that killed all MTB strains that were tested, and no emergence of phage resistance or antagonizing antibiotic effectiveness was observed. In another study, Dedrick et al. screened phage susceptible mycobacteria from 200 patients, and found 55 strains that could be killed by one or more phages (Dedrick et al., 2022). Among these positive hits, 20 selected patients were administered with strain-related phages through intravenous route and/or inhalation route and were monitored for clinical and microbiologic responses and adverse events. Physical improvements were observed in 11 patients, and no phage-related adverse reactions or phage resistance were reported in any of these 20 patients. Neutralizing antibodies were found in eight patients, but were not completely associated with the patients that did not show favorable responses (Dedrick et al., 2022).

In addition to these pre-clinical trials, a couple of case reports were also published. Dedrick et al. reported a compassionate use of phage therapy for an 81-year-old immunocompetent patient with *M. abscessus* lung disease (Dedrick et al., 2021). A three-phage cocktail, same as the one used for the 15-year-old patient as described earlier, was intravenously administrated to the patient twice daily for 6 months. Within the first month, a ten-fold drop in the *M. abscessus* sputum load was observed, and no adverse effects were detected. However, *M. abscessus* counts rebounded after 2 months post phage initiation, and reached the pretreatment level after 6 months, although posttreatment *M. abscessus* isolates remained sensitive to two of the three phages within the cocktail. This increase in bacterial counts was subsequently found to be associated with the increase in the patient’s antibody levels. This suggested that the induction of the antiphage neutralization antibody was to be the main reason for this failure. In another case report from Nick et al., a 26-year-old man with NTM lung disease was successfully treated using phage therapy (Nick et al., 2022). This patient was suffering from severe cystic fibrosis and chronic *M. abscessus* pulmonary infection and was treated with an intensive four-drug or five-drug antibiotic therapy, but still failed to obtain negative sputum cultures or to sustain lung function. The patient was declined for lung transplant by several hospitals, partly because of the unavailability of treatment options for his *M. abscessus* infection. A recommendation was then made for the patient to receive phage therapy for compassionate use. Two mycobacteriophages were engineered to enhance their bacteriolytic capacity and were intravenously administered to the patient as a therapeutic phage cocktail (phage BPsΔ*33*HTH_HRM10 and D29_HRM^GD40^). As a result, the genetic diversity of *M. abscessus* was reduced, and no increased resistance to antibiotics or phages was determined. Although anti-phage neutralizing antibody to one phage was detected, it did not affect the clinical improvement of the patient. In addition, the phage therapy was well tolerated, and no phage-related adverse events were reported. Due to the success in *M. abscessus* control, the patient received lung transplantation 1 year after the initiation of phage therapy, and experienced an uncomplicated recovery (Nick et al., 2022).

### 4.3. Perspectives in mycobacteriophage therapy

Since there are limited case reports on the successful treatment of mycobacterial infection with phages, it is difficult to conclude the efficacy of mycobacteriophage therapy. Furthermore, there are also failures being reported in using phages mainly due to host immune responses (Dedrick et al., 2021). Nevertheless, this does not hide the value of the two successful cases, which have actually shed light on a promising strategy for fighting against multi-drug resistant mycobacterial strains, albeit some improvements are still needed to tackle the issues that may be associated with phage therapy execution.

Each phage isolate has its particular host range, which is why it is essential to personalize phages for each patient. In addition, since phage efficacy could vary in *in vitro* and *in vivo* trials, monophage therapy may lead to modest outcomes (Chan et al., 2013). Thus, it is recommended to select two or more phages for each patient and to formulate them into a cocktail for enhanced efficacy. It’s important to keep in mind that bacteria can evolve resistance to phages by modifying or hiding phage receptors, changing the structure of polysaccharides, destroying viral DNA, or using other molecular mechanisms (Labrie et al., 2010). However, phage cocktails can also reduce the probability for the development of phage resistance. In recent years, many studies have focused on mycobacteriophage cocktails (Kalapala et al., 2020; Guerrero-Bustamante et al., 2021; Johansen et al., 2021), and the reported findings have vital implications for mycobacteriophage therapy development.

In fact, phage-resistant mutants are usually associated with bacteria-fitness costs, sometimes resulting in higher susceptibility to antibiotics (Senhaji-Kacha et al., 2021). Therefore, phage-antibiotic combination may be an optimal therapeutic idea, considering that phages are also natural enhancers of antibiotics. Some phages are able to break or inhibit the formation of biofilms that are generated by host bacteria, so antibiotics can access bacterial cells unencumbered (Wright et al., 2009). Several studies have confirmed the effects of the phage-antibiotic combination strategy on the biofilms of different pathogenic bacteria, including *Acinetobacter baumannii*, *Staphylococcus aureus*, and *P. aeruginosa* (Erol et al., 2023; Kebriaei et al., 2023; Racenis et al., 2023; Taha et al., 2023). Apart from the phage-antibiotic combination, phages also combine with other molecules, such as chlorine, xylitol, or iron antagonizing molecules to cause bacterial death by inhibiting the formation of biofilms (Chhibber et al., 2013; Zhang and Hu, 2013; Kaur et al., 2015). This further indicated the potential of the phage-molecule combination strategy. In addition to their effects on biofilms, some phages can change the structure of the host cell envelope to facilitate the entrance of antibiotics. For instance, the expression of the *gp39* gene from mycobacteriophage SWU1 can confer the host with increased cell wall permeability, thereby making host cells more susceptible to several antibiotics, including rifampicin, erythromycin, and vancomycin (Li et al., 2016).

For most cases of inefficient phage therapy, the induction of neutralizing antibodies is the major cause (Diacon et al., 2022). One way to protect against neutralizing antibodies is by encapsulating phages with liposomes (Singla et al., 2016). Encapsulated phages are less likely to be targeted by host’s immune system. They are also protected from possible inactivation by the stomach acidic pH. The encapsulation strategy could also prolong drug effects due to slower release (Colom et al., 2015; Otero et al., 2019). More importantly, liposomal encapsulation facilitates access to intracellular bacterial variants by encapsulated phages entering macrophages. Nieth et al. reported that the liposomes-encapsulated phage λeyfp and mycobacteriophage TM4 have better efficiency in entering eukaryotic cells than free phages (Nieth et al., 2015). However, it is still unclear if phage encapsulation within liposomes could prevent the acquisition of antibody-mediated neutralization over time. Another possible way to reduce antibody neutralization would be through the modification of virion glycans on the surface of a phage. Recently, Freeman et al. reported that virion glycans alter the production of phage-neutralizing antibodies in mice, suggesting that virion glycosylation may be a promising solution for mycobacteriophage therapy (Freeman et al., 2023). However, the immune responses in human may significantly differ from that in mice. The fact that neutralizing antibodies are highlighted as a major limitation to the application of mycobacterial phage therapy shows that further studies are required in this field, to prevent phage neutralization when phages are administered over an extended period of time.

Compared to antibiotics, phages are dynamic drugs that are accompanied by various advantages and disadvantages (Gordillo Altamirano and Barr, 2019). For example, phages can be administrated in low doses, owing to their reproduction ability. Moreover, they possess the ability to evolve alongside bacteria if phage resistance occurs. However, it is difficult to determine the pharmacokinetics of phages. In principle, phage adsorption, distribution, metabolism, and excretion can be computed *in silico*. However, due to the complexity that comes with human immunity and phage-bacterial interaction, it is impossible to propose a universal protocol for dosing and routes of phage administration without a large number of experimental trials (Caflisch et al., 2019). Since pathogenic mycobacteria are tedious to grow in terms of generation time, some animal disease models infected by fast-growing mycobacteria might be useful for exploring the impact of phage therapy. For example, the *Mycobacterium marinum* infected zebrafish model is promising, because *M. marinum* has a close genetic relationship with *M. tuberculosis*. However, it has the advantage that it can actually grow within a week (Medel-Plaza and Esteban, 2023). In addition, *M. marinum* has similar pathogenic mechanisms to *M. tuberculosis*, such as the formation of granuloma. However, the bottleneck to establish such a model is that there is no documented information about bacteriophages that are specific against *M. marinum* infections yet (Rybniker et al., 2006; Drakou et al., 2020; Medel-Plaza and Esteban, 2023). This might be due to an intracellular inhibition of phage replication in *M. marinum*, since shuttle phasmids based on some phages are able to transfect *M. marinum* (Rybniker et al., 2003, 2006). Although some progress has been made in phage pharmacology (Nang et al., 2023), and we now have more knowledge about treating infections with phage therapy than a century ago, the information about phage bioavailability, clearance rate, and binding affinity to both bacterial proteins and human plasma proteins is still not clear enough to establish a standard for using phage therapy. Understanding phage pharmacology is another key for effectively utilizing phage therapy in patients.

While pushing phage therapy forward, it is important that we are reminded of the lesson that emanated from continued antibiotic usage, which is, drug resistance. The application of phage therapy should be careful and appropriate, while the abuse of phages should be completely forbidden. Moreover, a variety of phage isolates are required to build large reservoirs for different mycobacterial strains, in case multiphage-resistance bacteria may emerge. Although many mycobacteriophages have been screened out, many of them are based on a specific host strain *M. smegmatis* mc^2^155, and their antibacterial spectra against other mycobacterial strains need to be determined, or be genetically modified (Hatfull, 2018). In addition, most mycobacteriophages isolated with *M. smegmatis* mc^2^155 are temperate phages (Hatfull, 2022), which only lyse bacteria at a certain period of their life cycle. If used in phage therapy, their bacterial lysing capacity may be weakened by the quick development of host immunity. In addition, bacterial cells can acquire antibiotic resistance or phage-encoded toxins from the lysogenic phages (Haaber et al., 2016; Gordillo Altamirano and Barr, 2019), and this might significantly enhance the virulence of pathogens. In contrast, virulent phages have higher potential in therapeutic development because they are more lethal to bacteria, thereby better imitating antibiotic drugs. Nevertheless, with genetic modification, lysogenic phages are also good candidates in phage therapy. A good example would be the 15-year-old case that we discussed earlier (Dedrick et al., 2019), where two genetically modified phages (BPs*33*ΔHTH-HRM10 and ZoeJΔ*45*) were used for phage therapy. These two phages are originally temperate phages (phage BPs and ZoeJ), but they attained the characteristics of being lytic and ability to eliminate target effectively pathogens through gene deletion.

## 5. Conclusion

Antimicrobial resistance is currently a major threat to the human society, and the MDR mycobacterial strains have led to increased patient morbidity and mortality. Novel alternative approaches, including mycobacteriophage therapy, are required to combat these MDR “superbugs.” Here, the potential use of mycobacteriophages against mycobacteria is reviewed. For the diagnosis of TB or NTM infections, the traditional gold standard is still the culturing method due to its high sensitivity and low cost, though it takes a long time to deliver results. The molecular diagnostic methods are fast and sensitive but are generally too expensive for middle- and low-income countries. In contrast, phage-based diagnostic tools are rapid, sensitive, and inexpensive to perform, but are not so specific to mycobacterial species. Nevertheless, since no perfect diagnostic methods have been developed so far, phage-based diagnostic tools are thus a good addition to the mycobacterial diagnosis toolbox.

Treatment of MDR mycobacteria is complex due to the inefficiency of conventional antibiotic therapies. Since phages have the intrinsic ability to lyse bacterial cells, they are considered to be natural alternatives to antibiotics. Although the clinical cases that have been reported yet are not many enough, phage therapy has its unique advantages. The dynamic dosing, specific host range, co-evolution ability with host bacteria, and low toxicity to human cells have offered phage therapy with the high potential of being applied clinically. However, more effort is still required to break down the bottlenecks in phage therapy, such as phage neutralization and low *in vivo* efficacy. Nevertheless, phage therapy has opened up a new gate for possibly escaping the rising antimicrobial resistance issue and could be considered a realistic alternative in clinical practice.

## Author contributions

XO: Writing – original draft, Writing – review & editing. XL: Writing – original draft, Writing – review & editing. JS: Writing – review & editing. HW: Writing – review & editing. SW: Writing – review & editing. RF: Writing – review & editing. ZL: Writing – review & editing. NS: Writing – review & editing.

## Footnotes

^1^ https://phagesdb.org
